# Simultaneous Holographic Molecular Binding Assays with Internal Calibration Standards

**DOI:** 10.1021/acs.langmuir.5c04611

**Published:** 2026-01-15

**Authors:** Kaitlynn Snyder, Andrew D. Hollingsworth, Fook Chiong Cheong, Rushna Quddus, David G. Grier

**Affiliations:** † Department of Physics and Center for Soft Matter Research, 5894 New York University, New York, New York 10003, United States; ‡ Department of Chemistry, New York University, New York, New York 10003, United States; § Adolph Merkle Institute, 311305 University of Fribourg, Fribourg CH-1700, Switzerland

## Abstract

Holographic molecular binding assays detect macromolecules binding to colloidal probe beads by monitoring nanometer-scale changes in the beads’ diameters with holographic microscopy. Measured changes are interpreted with Maxwell Garnett effective-medium theory to infer the surface coverage of analyte molecules and therefore to measure the analyte concentration in solution. We demonstrate a multicomponent holographic binding assay that tests for immunoglobulin G (IgG) using two different types of functionalized probe beads and provides internal negative controls using inert reference beads. The three label-free measurements are performed simultaneously and yield consistent results for the concentration of the analyte. Negative controls are validated by performing the same test on a solution of alcohol dehydrogenase (ADH), which has a similar molecular weight to IgG but does not bind to the probe beads’ binding sites. To assess and mitigate run-to-run variations that might affect the assay’s accuracy and reproducibility, we introduce a class of inert reference beads whose diameter and refractive index serve as standards for quantitative holographic microscopy measurements and whose polymer brush coating resists macromolecular binding. We characterize the reference beads’ coating by introducing a general all-optical method to measure the grafting density of the polymer brush. This measurement also yields a value of (1.308 ± 0.004) nm^3^ kDa^–1^ for the specific volume of poly­(ethylene oxide). This proof-of-concept demonstration of simultaneous independent holographic binding assays can be generalized into a platform for multiplexed testing.

## Graphical abstract

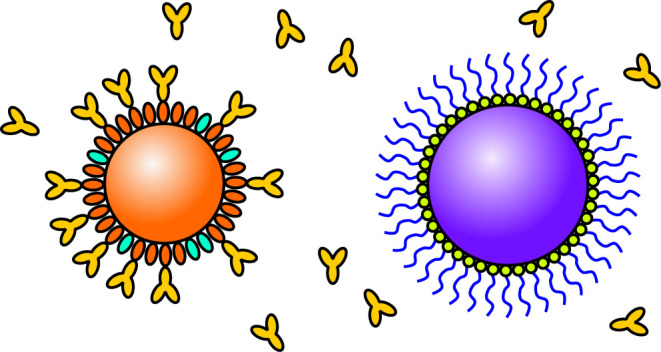

## Introduction

Holographic molecular binding assays^1–3^ use Total Holographic Characterization (THC)^4^ to detect molecules binding to the surfaces of suitably functionalized colloidal probe beads and thus to infer the concentration of those target molecules in solution. THC is a label-free particle-characterization method that records and analyzes holograms of individual colloidal particles in their native environment. When applied to micrometer-scale spheres, this analysis yields each bead’s diameter, *d*_p_, with nanometer precision and therefore can detect changes in diameter due to analyte molecules accumulating on the surface.^1,2,4,5^ THC simultaneously resolves each bead’s refractive index, *n*_p_, with part-per-thousand precision.^5,6^ The refractive index helps differentiate different classes of probe beads by their composition and therefore is useful for developing simultaneous bead-based binding assays.

A holographic binding assay compares THC results for ensembles of probe beads before and after incubation with samples that contain unknown concentrations of target molecules.^1–3,7^ Figure 1 represents a typical implementation of a holographic immunoassay^1,3^ in which antibodies (IgG) bind selectively to protein A molecules affixed to the surface of a probe bead (Figure 1(a)), but not to the surface of an inert reference bead (Figure 1(b)). Each recorded hologram, such as the example in Figure 1(c), yields a precise measurement of the diameters and refractive indexes of each bead in the field of view. THC measurements on thousands of beads are pooled into estimates for the population-averaged diameter, *d*_0_, before incubation, typically with nanometer precision. This value is compared with the mean probe-bead diameter after incubation, *d*_p_, to estimate the change in diameter,

1 $$\Delta d_{p}=d_{p}-d_{0}.$$

**Figure 1:**
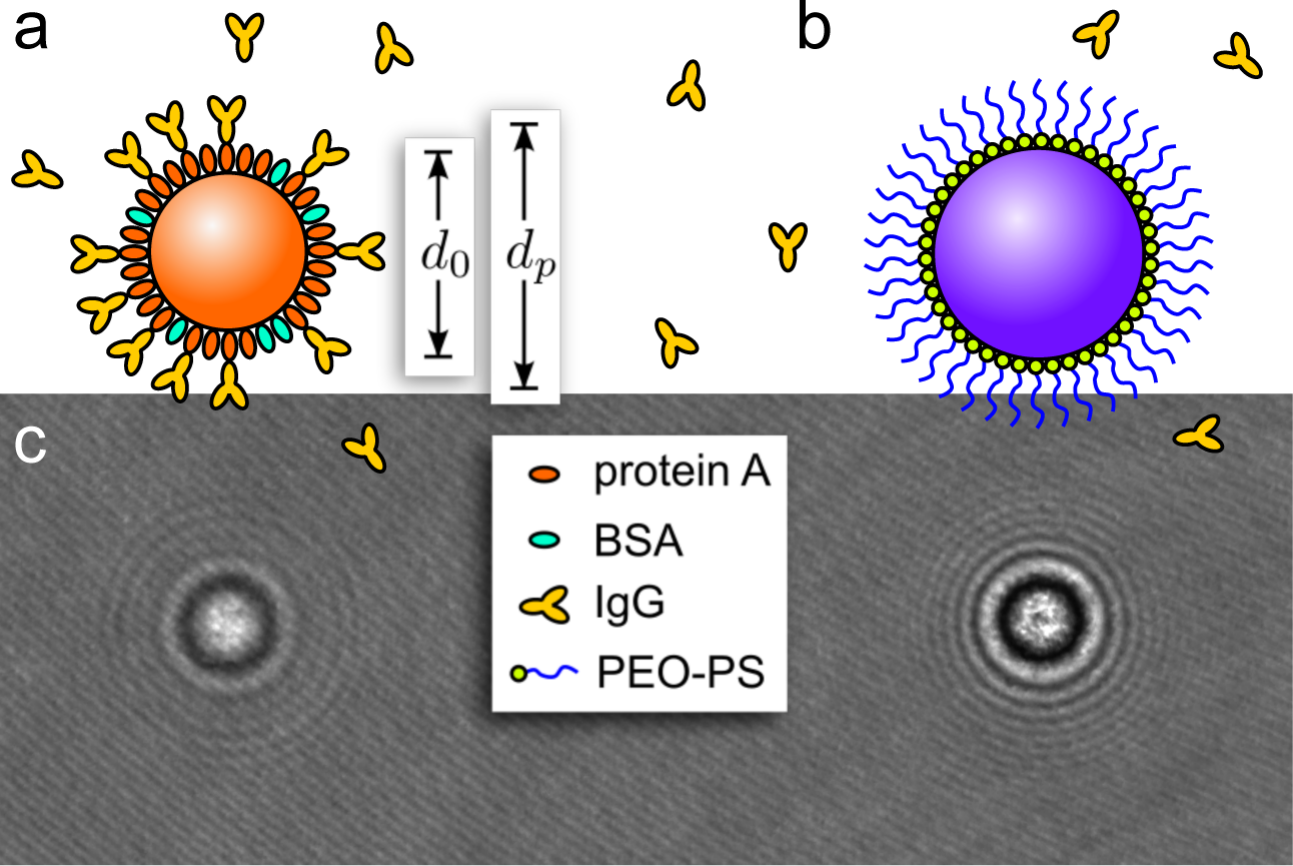
(a) A probe bead for a holographic immunoassay consists of a substrate bead functionalized with binding sites such as protein A. Bare spots on the surface are passivated with a blocking agent such as BSA. The hologram of such a bead encodes its starting diameter, *d*_0_. Target analytes such as IgG bind to the functionalized bead, increasing its measured diameter to *d*_p_. (b) The surface of a reference bead is covered with a dense PEO brush that inhibits macromolecular binding. Such inert beads serve as negative controls in binding assays. (c) In-line hologram of a silica-core probe bead (left) next to a PS-core reference bead (right). The beads are distinguishable after analysis by size and refractive index.

The observed diameter change is related to the thickness, *a*_c_, of the molecular-scale coating^2,3^ and therefore to the concentration, *c*, of the target analyte.^3^ THC does not rely on fluorescent labeling and thus eliminates the materials and processing required for fluorescence-based readout techniques. This in turn reduces the cost, complexity and time required to develop and deploy bead-based molecular binding assays.

Although THC measurements yield results with reproducibly high precision,^8^ run-to-run variations in instrumental performance^9^ can introduce systematic offsets in estimated parameters that reduce accuracy in Δ*d*_p_ and therefore degrade the sensitivity and limit-of-detection of the assay. Run-to-run variations can arise from systematic measurement errors, such as those due to variations between microfluidic cells or differences in the optical properties of the buffer medium. To assess and mitigate such systematic errors, we introduce a class of colloidal reference beads with distinctive optical properties that can be mixed into colloidal samples and will serve as reliable standards for the diameters and refractive indexes reported by THC. Ideally, characterization results for the reference beads will agree to within measurement precision for all runs. Significant deviations from the ground truth can be used to flag issues with specific THC measurements. Measured deviations in the reference beads’ properties also can be used to reduce run-to-run variation in the properties of other species in the sample.

Reference beads for holographic binding assays must resist nonspecific binding by proteins and other macromolecules in solution. The beads developed for the present study are sterically stabilized by a dense polymer brush grafted to their surfaces. We characterize the polymer brush by introducing general-purpose all-optical techniques to measure the optical specific volume of the stabilizing polymer molecules and the grafting density of polymers on the bead surface. We demonstrate the utility of reference beads for holographic assays by performing two independent holographic immunoassays for IgG using inert reference beads as an internal negative control. The success of this assay serves to validate the underlying effective-medium interpretation of holographic characterization data used to analyze the results. The proof-of-concept demonstration of multichannel parallel assays with internal negative controls advances the technology for label-free bead-based holographic binding assays toward future clinical applications.

## Experimental Section

### Holographic Molecular Binding Assays

Holographic molecular binding assays are performed by mixing an analyte solution with a test kit containing a dispersion of micrometer-scale colloidal probe beads and inert reference beads. The mixture is incubated to allow target analytes to bind to the probe beads. Samples of the dispersion are analyzed with Total Holographic Characterization before and after incubation to detect nanometer-scale changes in the beads’ diameters that could indicate specific binding of macromolecules to their surfaces. These results are then interpreted with effective-medium theory to estimate the concentration of target analyte molecules in the original solution.

### Multicomponent Assay Kit with Negative Controls

The test kit developed for this study is a colloidal dispersion that performs two independent assays for immunoglobulin G (IgG) and validates their results with internal negative controls. The dispersion consists of three types of colloidal beads: 1 μm-diameter polystyrene (PS) probe beads precoated with protein A (Bangs Laboratories, catalog no. CP02000, lot no. 14540);^3^ 1 μm-diameter silica probe beads (General Engineering & Research) custom coated with protein A; and 1.3 μm-diameter PS reference beads (Thermo Fisher Scientific, catalog no. 5130A, lot no. 172008) custom coated with poly­(ethylene oxide) (PEO). Protein A enables functionalized beads to bind antibodies to their surfaces.^3^ The PEO-coated PS beads are designed to inhibit macromolecular binding and therefore serve as negative controls for binding-induced changes in the functionalized test beads.

The PS test beads are coated with protein A binding sites by the manufacturer. The silica probe beads are functionalized with protein A using a PolyLink Protein Coupling Kit (Bangs Laboratories, catalog no. PL01N). The PEO-coated PS references beads are prepared according to the protocol described below. These latter beads not only lack specific binding sites, but are passivated by a dense polymer brush that sterically stabilizes their surfaces.

Probe beads and reference beads are dispersed at 1:1:1 stoichiometry in antibody binding buffer consisting of 50 mM sodium borate buffer prepared with boric acid (99.5%, Sigma-Aldrich, catalog no. B0394, lot no. SLBM4465 V) and NaOH (98%, Sigma-Aldrich, catalog no. S8045, lot no. 091M01421 V) in deionized water (18.2 MΩ cm, Barnstead Millipure). The buffer is adjusted to pH 8.2 with the addition of dilute HCl (38%, Sigma-Aldrich, catalog no. H1758).^10^ Bovine serum albumin (BSA) (Sigma-Aldrich, catalog no. A4503) is added at 0.01% w/v to inhibit nonspecific binding by blocking any bare regions on the surfaces of the probe beads. The overall concentration of beads is adjusted to 3 × 10^6^ beads/mL for compatibility with THC.

This test kit is designed to assess the performance of label-free holographic characterization measurements for simultaneous binding assays through multiple channels. Including two distinct assays for the same analyte allows us to assess the measurements’ quantitative consistency despite differences in the probe beads’ composition. Including inert reference beads enables us to validate the performance of those custom-synthesized beads as negative controls and also to quantify run-to-run variations that might affect the precision and accuracy of the associated assays.

Given these goals, the kit’s composition is designed for reproducibility and ease of use with idealized analyte solutions. Applications to real-world samples are likely to require a different choice of buffer, different choices for blocking agents, and additional excipients to maintain the assays’ selectivity and sensitivity. These choices also would carry over to multiplexed assays in which the different types of optically distinguishable probe beads are functionalized to bind to different analytes. All such elaborations are compatible with the label-free holographic detection technique, but are beyond the scope of the present study.

### Simultaneous Holographic Characterization of Probe Beads and Reference Beads

THC measurements are performed with a commercial instrument (xSight, Spheryx). Each measurement involves transferring 30 μL of the fluid sample into one of the reservoirs in a dedicated microfluidic chip (xCell8, Spheryx). The measurement proceeds automatically, with a selected portion of the fluid in the reservoir being transported through the instrument’s observation volume in a pressure-driven flow. THC analysis provides a record of the diameter, refractive index, and morphology of each particle in the measured sample, together with uncertainties in those values. THC can be applied to beads ranging in diameter from 500 nm to 10 μm dispersed in fluid media at concentrations ranging from 10^3^ beads/mL to 10^7^ beads/mL. Figure 2 shows the results from one such measurement, with each plot symbol representing the diameter, *d*_p_, and refractive index, *n*_p_, one particle in a stoichiometric mixture of probe beads and reference beads. A 1 μL volume of this sample yields results for roughly 1000 beads of each type, indicating an overall concentration of 3 × 10^6^ beads/mL. The data points in Figure 2 are colored by the density of observations, ρ­(*d*_p_, *n*_p_) and form three clusters, one for each population of particles. Regions of interest indicated in Figure 2 identify each of these three populations and are used to assess variations in the properties of those populations from run to run.

**Figure 2:**
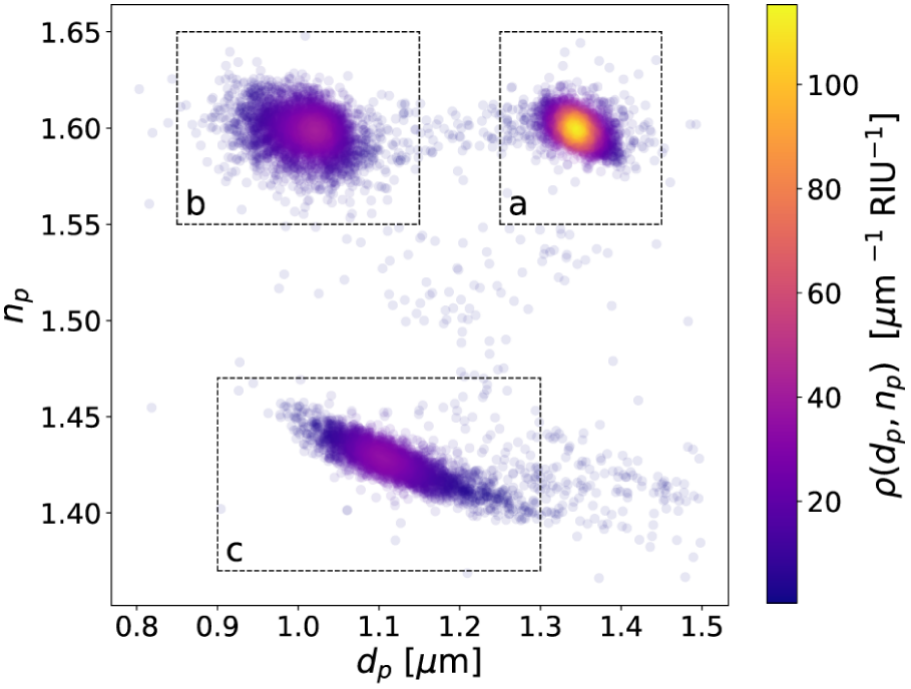
Holographic particle characterization data for a mixture of (a) reference beads, (b) immunoassay probe beads with PS substrates and (c) immunoassay probe beads with silica substrates, all in the same dispersion at an overall concentration of 3 × 10^6^ beads/mL. Each of the 11616 points represents the diameter, *d*_p_, and refractive index, *n*_p_, of a single particle. Points are colored by the density of observations, ρ­(*d*_p_, *n*_p_). Each bead type occupies a distinct region of the *d*_*p*_–*n*_p_ plane allowing several assays to be performed simultaneously.

THC measurements on bare PS substrate beads yields population-averaged values for the diameter, *d*_0_ = (1.3408 ± 0.0002) μm, and the refractive index, *n*_0_ = 1.6019 ± 0.0001. Consistent values are obtained when the beads are dispersed in DI water or in 1 nM NaCl. The uncertainty in the last digit is the standard error of the mean for the entire set of measurements, and reflects the precision with which changes in particle diameter and refractive index can be resolved.

The standard error in the mean diameter is much smaller than the standard deviation, σ_d_ = 0.028 μm, which reflects the 2% polydispersity in the beads’ underlying size distribution. Pooling independent measurements accounts for run-to-run variations that are specified by the instrument’s manufacturer to be as large as 5 nm in the diameter and 0.003 in the refractive index.

### Holographic Analyte Binding Measurements

Label-free holographic binding assays are performed at an analyte concentration of 2.5 mg mL^–1^ to mimic testing conditions at the lower end of the physiologically relevant range of concentrations.^3,11^ The same concentration of ADH is used as a negative control to both to demonstrate that the passivated probe beads are free from nonspecific binding and also to demonstrate that the reference beads are inert.

The assay is initiated by adding a 4 μL aliquot of the analyte solution to 196 μL of the multicomponent assay kit dispersion. Adding the analyte to the test solution effectively dilutes the analyte to a concentration of 50 μg mL^–1^. The system is allowed to incubate at room temperature for 45 min under gentle agitation on a rotary platform (MS 3 basic, IKA). Three 30 μL samples are then transferred into separate reservoirs on microfluidic chips for THC analysis in triplicate. Each such measurement yields a distribution of holographically detected particle properties such as the example in Figure 2.

The three types of beads in the mixed dispersion give rise to distinctive clusters of points in the *d*_p_–*n*_p_ plane, which allows for straightforward data segmentation. The dashed boxes in Figure 2 reflect the sampling regions used for this study. The data clusters associated with each bead type are then compared from run to run with Kolmogorov–Smirnov tests to ensure consistency. If the replicated measurements are found to be consistent, the results for each bead type are pooled. This procedure is followed for both control and analyte incubation experiments. The mean diameter, *d*_p_, then is calculated for each bead type, along with the standard error in the mean. These values finally are compared with values obtained for the same beads in the same buffer before incubation to obtain the differences, Δ*d*_p_, for each bead type. Observed diameter changes can be used to assess whether the target analyte was present in the sample and to infer its concentration.^1,3^

### Reference Bead Functionalization

As illustrated schematically in Figure 1(b), the reference beads introduced in this study for THC measurements consist of monodisperse polystyrene (PS) spheres whose surfaces are sterically stabilized against physisorption of macromolecules by a dense coating of poly­(ethylene oxide) (PEO). Polystyrene beads are suitable substrates both because of their commercial availability and also because their high refractive index minimizes the influence of any unintended adsorbates on the their light-scattering properties,^2^ which is a desirable feature for reference particles.

The protocol used to coat PS beads with a dense brush of PEO is described in ref. 12. Commercial PS beads are swollen by adding tetrahydrofuran (THF) to their aqueous dispersion and are incubated with a PS-*b*-PEO diblock copolymer. The hydrophobic PS blocks dissolve in the swollen PS spheres and are anchored in place by evaporating the THF to deswell the beads. This protocol leaves the hydrophilic PEO blocks exposed on the beads’ surfaces.

Reference beads are prepared by dispersing 1.3 μm-diameter PS beads (Thermo Fisher Scientific, catalog no. 5130A, lot no. 172008) at 10% w/v in a solution composed of 140 μL poly­(styrene-*b*-ethylene oxide) (PS-*b*-PEO) solution (catalog no. P1807A-SEO, Polymer Source, Inc.), 90 μL DI water, and 160 μL THF at room temperature. The block copolymer consists of 3.8 kDa PS covalently linked to 34 kDa PEO. This mixture is placed on a horizontal shaker at 900 rpm for 1.5 h. After incubation, THF is removed from the solution by evaporation at room temperature over the course of 2 h. Excess polymer is removed by washing the particles three times in deionized water. Each washing cycle involves centrifuging the dispersion at 6500 rpm for 5 min to concentrate the beads, removing the supernatant, and redispersing the beads in deionized water.

The tethered PEO block has a contour length of roughly l=216nm , assuming an incremental length of 0.28 nm/monomer^13,14^ and an expected radius of gyration of *R*_g_ = 9.1 nm.^15^ The stabilizing properties of the molecular brush formed by these molecules depend on the molecules’ specific volume and their grafting density on the surface, both of which can be measured optically.

### Holographic Measurement of PEO Grafting Density on Reference Beads

As depicted schematically in Figure 1(b), our reference beads are coated with a dense brush of PEO. The quality of their functionalization is gauged by the grafting density, Γ_c_, of PEO molecules on the surface of the PS bead. Here, we introduce a method to measure the grafting density based on THC measurements of the beads’ population-average diameter, *d*_p_, and refractive index, *n*_p_, before and after functionalization. The beads’ holographically measured properties change due to the addition of a molecular scale coating in a manner that is predicted by effective medium theory.

The observed increase in the beads’ diameter, Δ*d*_p_, generally does not correspond to twice the coating thickness, *a*_c_, because the refractive index of the coating, *n*_c_, generally does not match that of the substrate bead, *n*_0_. Instead, numerical studies of the optical properties of coated spheres^2^ show that Δ*d*_p_ is related to *a*_c_ by

2a $$\Delta d_{p}=2a_{c}\frac{n_{c}-n_{m}}{n_{0}-n_{m}}.$$

The coating consists of macromolecules of intrinsic refractive index *n*_1_ at volume fraction ϕ_c_ interspersed with the fluid medium at refractive index *n*_m_. The effective refractive index of the coating then follows from eq 10,

2b $$n_{c}\left(\phi_{c}\right)=n_{m}\sqrt{\frac{1+2\phi_{c}L\left(m_{1}\right)}{1-\phi_{c}L\left(m_{1}\right)}},$$

where *L*(*m*) is defined in eq 11 and *m*_1_ = *n*_1_/*n*_*m*_ is the refractive index of a single macromolecule relative to that of the medium. The macromolecules’ volume fraction depends on their grafting density,

2c $$\phi_{c}=\frac{v_{s}\Gamma_{c}}{a_{c}},$$

where *v*_*s*_ is the optical specific volume of the grafted PEO molecule. An all-optical method to measure *v*_*s*_ is presented in the next section.

Through eq 2, a THC measurement of Δ*d*_p_ imposes three constraints on four characteristics of the coating: Γ_c_, *a*_c_, *n*_c_ and ϕ_c_. This set of relationships can be expressed as an overall constraint on the grafting density, Γ_c_, and layer thickness, *a*_c_, conditioned on the measurement of Δ*d*_p_:

3 $$\Gamma_{c}\left(a_{c}|\Delta d_{p}\right)=\frac{x}{v_{s}L\left(m_{1}\right)}\frac{a_{c}^{2}+xa_{c}}{\frac{3}{4}a_{c}^{2}+xa_{c}+x^{2}},$$

where

4 $$x=\frac{1}{4}\left(\frac{n_{0}}{n_{m}}-1\right)\Delta d_{p}$$

is the scaled diameter shift.

Equation 5 applies without modification when macromolecules are grafted or physisorbed directly to the surface of the substrate bead. The PS block of the copolymer brush, however, is embedded in the substrate bead. This increases the bead’s diameter by an amount that depends on the grafting density:

5 $$\Delta d_{0}\left(\Gamma_{c}\right)\approx 3\frac{m_{\mathrm{PS}}}{\rho_{\mathrm{PS}}}\Gamma_{c},$$

where *m*_PS_ = 3.8 kDa is the mass of the PS block and ρ_PS_ = 1.05 × 10^3^ kg m^–3^ is the mass density of polystyrene. Equation 5 then can be solved for Γ_c_ self-consistently using

6 $$x=\frac{1}{4}\left(\frac{n_{0}}{n_{m}}-1\right)\left[\Delta d_{p}-\Delta d_{0}\left(\Gamma_{c}\right)\right].$$

Figure 3 presents Γ_c_(*a*_c_|Δ*d*_p_) for the 34 kDa PEO brush used to functionalize our reference beads, conditioned on THC measurements of Δ*d*_p_. THC measurements are performed in 1 mM NaCl solution for consistency with electrokinetic characterization measurements. Each measurement is performed seven times to minimize the influence of run-to-run instrumental variations and the results are pooled. This procedure yields Δ*d*_p_ = (3.3 ± 0.3) nm. The other material parameters in eq 5 are the refractive indexes *n*_1_ = 1.470 ± 0.005 and *n*_m_ = 1.340 ± 0.001, which are appropriate for PEO and water, respectively, at the wavelength of light used for THC, and *v*_s_ = (44.5 ± 0.1) nm^3^. The shaded region in Figure 3 reflects the uncertainties in these values. These results self-consistently account for an increase in the substrate bead diameter up to Δ*d*_0_ = (0.7 ± 0.1) nm due to incorporation of the PS blocks.

**Figure 3:**
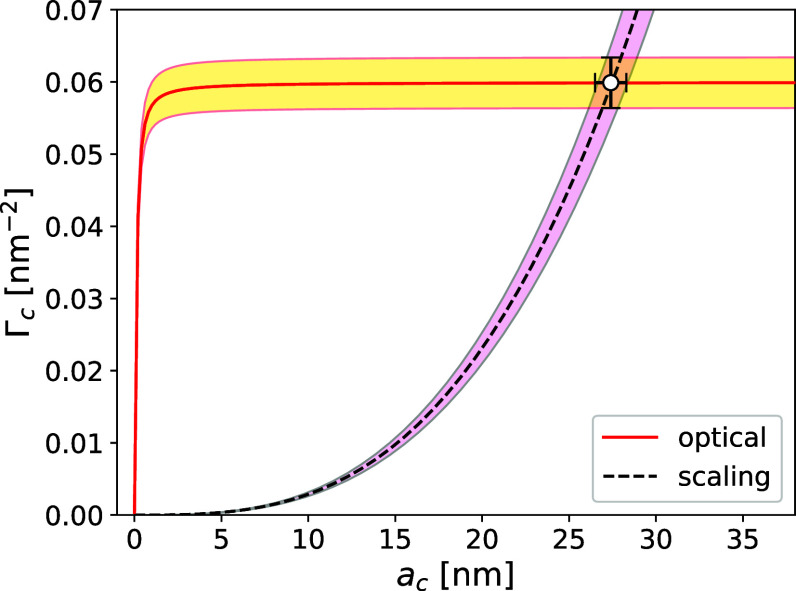
Solid (red) curve: values of the grafting density, Γ_c_, and coating thickness, *a*_c_, of PEO-grafted PS beads consistent THC measurements of the diameter increase, Δ*d*_p_, according to eq 5. Dashed (black) curve: scaling prediction from eq 9 for the thickness of a grafted polymer brush as a function of grafting density, including uncertainties in material parameters. Plot symbol: estimates for *a*_c_ and Γ_c_ assuming consistency between effective-medium theory and scaling theory.

The dashed curve in Figure 3 is the predicted scaling relationship,^16^

7 $$a_{c}\left(\Gamma_{c}\right)={\left(\frac{1}{6}l^{2}v_{s}\Gamma_{c}\right)}^{1/3},$$

between the thickness of a Gaussian polymer brush and its grafting density. Requiring consistency between this prediction and the optical constraint from eq 5 yields Γ_c_ = (0.060 ± 0.004) nm^–2^ for the grafting density and *a*_c_ = (27.5 ± 0.9) nm for the effective coating thickness. Comparable results for Γ_c_ have been reported for the same grafting procedure on similar beads using orthogonal measurement techniques.^12,15^

The estimated value of Γ_c_ is consistent with a mean separation between tethered PEO molecules of 4 nm, which is is smaller than the individual molecules’ radius of gyration, *R*_g_ = 9.1 nm. The coating therefore is sufficiently dense for the grafted polymers to form a brush. The separation also is smaller than the dimensions of typical target analytes for molecular binding assays, which contributes to the stability of the PEO-coated reference beads against physisorption.

### Optical Measurement of the Specific Volume of PEO

The optical specific volume of a polymer is the volume, *v*_s_, that a single molecule occupies in solution as gauged by the molecule’s influence on the solution’s optical properties.^17^ This value does not include solvation effects and is largely independent of the molecule’s conformation. The optical specific volume is especially useful for the insights it offers into the relative contribution of each molecular species to the refractive index of a heterogeneous medium. In that respect, it is complementary to standard d*n*/d*c* analysis,^18^ in which the refractive index increment is used to measure molecular weight rather than molecular volume.

The measured refractive index of a polymer solution, *n*, is related to the volume fraction, ϕ, of polymer molecules in solution through Maxwell Garnett effective-medium theory:^19^

8a $$L\left(\frac{n}{n_{m}}\right)=\phi L(\frac{n_{1}}{n_{m}}),$$

where *n*_1_ is the intrinsic refractive index of the polymer, *n*_m_ is the refractive index of the fluid medium and

8b $$L\left(m\right)=\frac{m^{2}-1}{m^{2}+2}$$

is the Lorentz–Lorenz factor. The volume fraction, in turn, is proportional to the polymer’s concentration, *c*,

8c $$\phi =cv_{s},$$

where *v*_s_ is the specific volume of a single polymer molecule. Equation 10 therefore describes a protocol for measuring the optical specific volume of a polymer based on measurements of *n*(*c*).

The data in Figure 4 were obtained for solutions of poly­(ethylene glycol) (PEG) of various molecular weights in 5 mM sodium phosphate buffer at pH 7. The refractive index of each solution is measured with an Abbe refractometer (Edmund Optics, model 52–975) at a vacuum wavelength of 589 nm and is used to estimate the volume fraction of polymer in solution according to eq 10 using *n*_m_ = 1.3325 ± 0.0005 and *n*_1_ = 1.466 ± 0.004.^20–22^ The five data sets in Figure 4(a) show results for five different mean molecular weights: 1.5 kDa (Fluka Analytical, catalog no. 81210), 4 kDa (bioWorld, catalog no. 714224), 6 kDa (Millipore, catalog no. 528877), 8 kDa (bioWorld, catalog no. 705631) and 20 kDa (Fluka Analytical, catalog no. 95172). Each sample is prepared at 40% w/v and is sequentially diluted by factors of 2 to obtain ϕ­(*c*). The slope of each linear trend yields *v*_*s*_ for PEG at the associated molecular weight. We assume that the same value for the optical specific volume can be used to interpret optical measurements at other wavelengths, including THC measurements.

**Figure 4:**
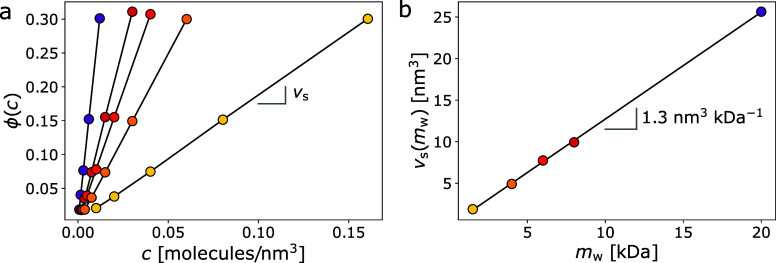
(a) The volume fraction, ϕ­(*c*), occupied by PEG molecules in solution as a function of concentration, *c*, for five molecular weights, estimated from refractive index measurements using eq 10. The slope of each line gives the optical specific volume, *v*_s_ for each molecular weight, *m*_w_. (b) The optical specific volume of each PEG molecule as a function of molecular weight obtained from (a). The slope, 1.3 nm^3^ kDa^–1^ can be used to estimate *v*_s_ for other molecular weights.

The refractometry data plotted in Figure 4(b) confirm that the specific volume of PEO, *v*_s_, scales linearly with molecular weight, *m*_w_, with a slope d*v*_s_/d*m*_w_ = (1.308 ± 0.004) nm^3^ kDa^–1^. This fundamental property of PEO appears not to have been reported previously and is useful for estimating the specific volume of PEG and PEO at arbitrary molecular weights. Applying it to the PS-*b*-PEO diblock copolymer used to functionalize the reference beads in this study yields a value for the specific volume of the 34 kDa PEO block: *v*_s_ = (44.5 ± 0.01) nm^3^.

### Electrokinetic Characterization of Reference Beads

Electrokinetic characterization measurements provide an independent check of the all-optical measurements of the thickness of the PEO brush on the PS reference beads. These measurements are performed using a Zetasizer Nano (Malvern Panalytical) both before and after functionalization with PEO. Unlike THC measurements, which can be performed in any fluid medium, electrokinetic characterization requires the particles to be suspended in 1 mM NaCl solution. The bare beads yield a zeta potential, ζ_p_ = −81 mV, and electrophoretic mobility, μ_e_ = −6.3 μm cm V^–1^ s^–1^, that are consistent with expectations^23^ for polystyrenesulfonate beads. The corresponding values after coating with PEO, ζ_p_ = −2.1 mV and μ_e_ = −0.16 μm cm V^–1^ s^–1^, suggest a forty-fold reduction in effective surface charge, which correspondingly reduces the beads’ interactions with charged species in solution. Fitting these data to the standard model for electrophoretic mobility of “fuzzy” colloids^15,24^ yields a brush thickness of *a*_c_ = (34 ± 2) nm.^15^ This value is larger than the result obtained from optical measurements, (27 ± 1) nm, which may reflect differences in the assumptions underlying the models used to analyze the two types of measurements. Most notably, eq 2 does not account for the radial density gradient in the polymer brush and therefore tends to underestimate *a*_c_.

## Results and Discussion

### Optical Specific Volume of PEO

The technique introduced in the Experimental section for measuring the optical specific volume yields

9 $$\frac{dv_{s}}{dm_{w}}=\left(1.308\pm 0.004\right)\;{\mathrm{nm}}^{3}\;{\mathrm{kDa}}^{-1}$$

for the differential specific volume of PEO and PEG. When multiplied by the molecular weight, this value represents the intrinsic volume of the macromolecule. Specifically, this is the effective volume of the molecule based on its interaction with light, independent of its conformation or interactions with the solvent.

### PEO Grafting Density on Reference Beads

Holographic measurements of the diameter of PEO-coated beads are combined with refractometry measurements of the macromolecular specific volume to obtain a constraint condition between the grafting density of macromolecules and the thickness of the macromolecular coating. Requiring consistency with theoretical predictions from eq 9 yields the grafting density of PEO on the reference beads prepared for this study:

$$\Gamma_{c}=\left(0.060\pm 0.004\right)\;{\mathrm{nm}}^{-2}.$$ 10

This grafting density is consistent with a mean polymer separation of 4 nm, which is smaller than the radius of gyration of the individual polymers, *R*_g_ = 9.1 nm. This indicates that the PEO molecules elongate to form a polymer brush on the reference bead surface. Crucially, this polymer separation is smaller than the dimensions of the target analyte molecules used in this study, providing further support for the inertness of these PEO reference beads.

### Holographic Detection of Analyte Binding

Typical data for a label-free bead-based holographic molecular binding assay are plotted in Figure 5 and are summarized in Table 1. The top row in Figure 5 shows pooled probability densities for the particle diameters, ρ­(*d*_p_), for each of the three bead types. Distributions are normalized to unity peak height to facilitate comparison of the shapes of the distributions. Results are shown for the stock beads before incubation (shaded) and for the beads after incubation with IgG and with ADH. The middle and bottom rows show changes in the distributions after incubation. The PS probe beads show a clear response to incubation with IgG and very little response to incubation with ADH. Silica probe beads respond more strongly to IgG and just as weakly to negative control measurements with ADH. The reference beads developed for this study show no response either to IgG or to ADH.

**Figure 5:**
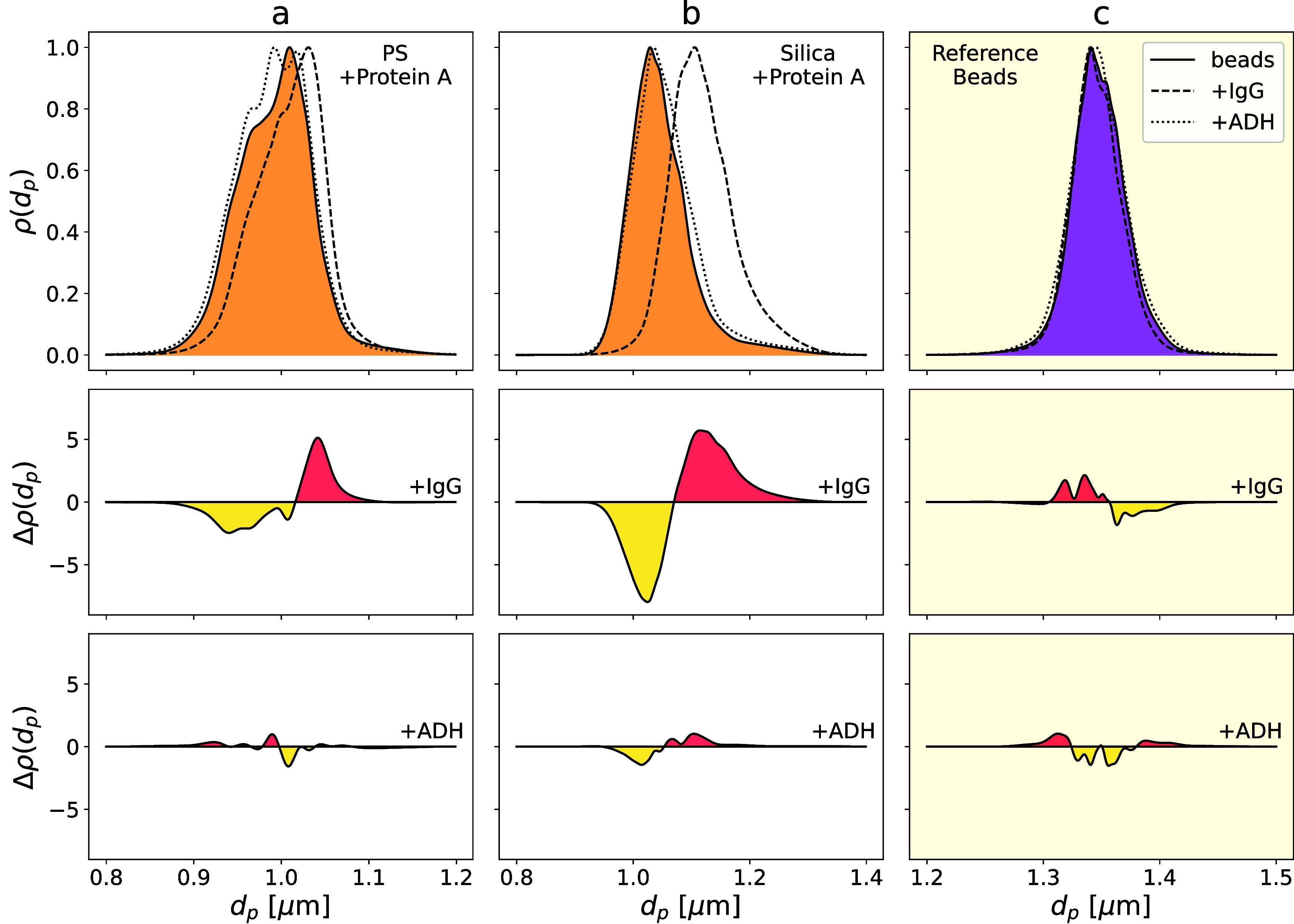
(top row) THC measurements of the diameter distribution, ρ­(*d*_p_), for (a) PS probe beads, (b) silica probe beads and (c) PS reference beads before and after incubation with IgG or ADH. (middle row) Differences, Δρ­(*d*_p_), between the distributions before and after incubation distributions with 50 μg mL^–1^ IgG showing clear positive responses from the probe beads and no response from the reference beads. (bottom row) Control measurements of Δρ­(*d*_p_) with 50 μg mL^–1^ ADH show no response from either probe or reference beads.

**Table 1:** Mean Bead Diameter, *d*_0_, and Observed Increase in Bead Diameter, Δ*d*_p_, after Incubation with 50 μg mL^–1^ of Either IgG or ADH^a^

		Δ*d*_p_ [nm]	
Bead Type	*d*_0_ [μm]	IgG	ADH
PS Probe	0.9920(6)	16(1)	–2(1)
Silica Probe	1.0436(8)	71(2)	8(2)
PS Reference	1.3476(4)	–2(1)	0(1)

a Probe beads functionalized with protein A respond to IgG whereas PEO-coated reference beads do not. Negative control measurements with ADH elicit no response from any of the beads.

### Simultaneous Independent Molecular Binding Assays with Integrated Negative Control

Figure 6 reports the results of an immunoassay performed with the multicomponent test kit developed for this study. This test kit performs two independent assays for antibodies such as IgG, and provides internal negative controls using the PEO-coated inert reference beads. The three classes of beads are codispersed in the same test kit and therefore are brought into equilibrium with the same analyte solution. The mean diameter, *d*_p_, of each population of beads is determined using Total Holographic Characterization on statistical samples of 1000 to 2000 beads of each type both before and after incubation. These measurements are repeated in triplicate and the results are pooled. The (orange) box-and-whisker plots in Figure 6 report the population-averaged values of *d*_p_ for control measurements before incubation, negative control measurements with ADH, and test measurements with IgG for each of the three classes of beads. These assays report a positive response with IgG in both the PS test beads (Δ*d*_p_ = (16 ± 1) nm) and the silica test beads (Δ*d*_p_ = (71 ± 2) nm).

**Figure 6:**
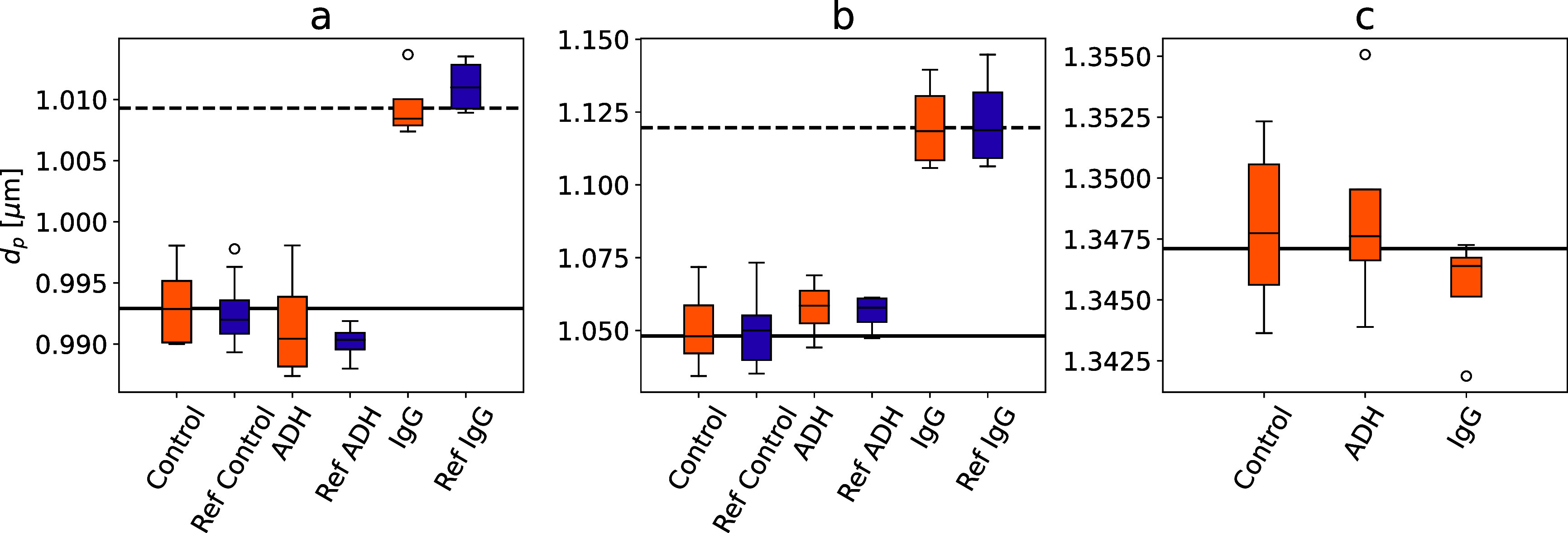
Box-and-whisker plots showing bead diameter distributions in multicomponent holographic immunoassays before (orange) and after (purple) correcting for run-to-run variations with reference-bead standards. (a) PS test beads functionalized with protein A. (b) Silica test beads functionalized with protein A. (c) PEO-passivated PS reference beads. Each result is pooled from at least three replicated measurements and includes holographic characterization data from at least 5000 beads. Control measurements are performed on the assay kit before incubation with analyte solutions.

The observed sensitivity of the PS-bead assay agrees with previously reported results^3^ for this type of test bead at this analyte concentration, although the lower intrinsic polydispersity in properties of the particles used for this study, improved protocols for test-bead functionalization, mitigation of nonspecific binding with blocking agents and increased sample size provides a 4-fold reduction in uncertainty. The silica test beads have a 4-fold larger response in the same analyte. This difference can be attributed to the lower refractive index of the silica substrate beads and provides an experimental confirmation of the predicted dependence of an assay’s response on the substrate bead’s composition.^2^

As reported in Figure 6(c), the inert PEO-coated PS beads show no statistically significant response to either ADH or IgG, confirming their utility as built-in negative controls for holographic binding assays. From run to run, however, the measured values of the mean bead diameter can vary by as much as 5 nm, possibly due to systematic effects such as manufacturing variations in the microfluidic cells’ optical properties. Such systematic effects might also influence the results for the probe beads. To assess and potentially mitigate such effects, we compute the deviation, δ*d*_p_, of the measured diameter of the reference beads from a nominal ground-truth value calculated by pooling data from 17 separate holographic characterization measurements comprising a total of 14,476 particles. We then subtract the same deviation from the measured diameters of the other types of beads in the same run. This naive correction yields the (purple) reference-corrected results in Figure 6. For this set of measurements, reference-based corrections do not significantly change the mean bead diameters obtained by pooling replicated measurements. This suggests that errors are dominated by statistical uncertainty rather than systematic instrumental errors. The presence of the reference beads helps to validate the assay by minimizing the possibility that the detection of IgG resulted from a false positive reading or that the absence of a signal in the ADH assay resulted from a false negative reading.

The two independent assays for IgG in the multicomponent test kit should report consistent results for the concentration of the target analyte despite the 4-fold difference in their responses to analyte binding. In both cases, the thickness, *a*_c_, of the molecular coating formed by bound IgG should be comparable to the size of a single antibody. The coating’s effective refractive index, *n*_c_, depends on the areal density of binding sites on the beads’ surfaces and their fractional occupation in equilibrium given the analyte’s concentration.^3^ If we assume that the two types of probe beads have comparable areal densities of binding sites, then eq 2 suggests that

11 $$a_{c}=\frac{1}{2}\frac{n_{0}-n_{m}}{n_{c}-n_{m}}\Delta d_{p}$$

should provide an estimate for the true coating thickness and therefore should have the same value in both assays. The results,

12a $$a_{c}\left(\mathrm{PS}\right)=\left(13\pm 2\right)\;\mathrm{nm}$$

12b $$a_{c}\left(\mathrm{silica}\right)=\left(15\pm 3\right)\;\mathrm{nm}$$

indeed are consistent with each other and are consistent with the actual dimensions of IgG. Consistency between simultaneous assays based on different types of substrate beads supports the conclusion from earlier reports^1–3^ that effective-medium analysis of holographic bead-based binding assays accurately models macromolecular binding. This observation also builds confidence that parallel and multiplexed holographic binding assays can provide accurate analyte concentrations regardless of the composition of the substrate beads.

## Conclusions

Total Holographic Characterization (THC) measures the diameters of micrometer-scale colloidal beads with the precision and accuracy needed to detect nanometer-scale macromolecules binding to their surfaces. The standard analysis for THC^4^ treats each bead as a homogeneous sphere. THC data for inhomogeneous particles such as coated beads can be interpreted with effective-medium theory^2,3,19,25^ to obtain information about properties of the coating. The present study uses this all-optical technique to achieve two goals: (1) to measure the grafting density of a poly­(ethylene oxide) (PEO) brush on the surface of polystyrene (PS) beads, and (2) to demonstrate a simultaneous label-free immunoassay using the PEO-coated PS beads as internal negative controls. The techniques developed for this proof-of-concept demonstration can be applied in any context where macromolecular coatings form on colloids and can be particularly useful for assessing properties of the coatings.

The two independent assays implemented by the multicomponent test kit both use protein A to bind IgG to the test beads’ surfaces. THC clearly distinguishes the two different types of test beads from each other and from the reference beads both by size and also by refractive index. The two tests both respond strongly to the presence of IgG in the analyte solution and show no significant response to negative control measurements performed with ADH. By performing parallel independent assays for the same analyte, we are able to verify that the quantitative assay results are consistent with each other when interpreted with effective-medium theory. This consistency provides additional validation for the overall use of effective-medium theory to interpret THC results of coated spheres. More generally, the test kit used for a holographic molecular binding assay can combine multiple classes of holographically distinguishable beads that are each functionalized for distinct target molecules.

Including inert reference beads in the test kits provides an internal assessment of run-to-run variations in differential and replicated THC measurements. In reporting the grafting density of the stabilizing polymer brush on these reference beads, the present study also reports the differential specific volume of poly­(ethylene oxide) (PEO), which appears not to have been reported previously. The determined grafting density reveals a dense coverage of PEO with the polymer spacing smaller than the dimensions of the analyte molecules used in this study. This further supports the use of these densely grafted PEO reference beads as an inert control for holographic binding assays. In addition to detecting systematic errors due to instrumental run-to-run variations, THC analysis of the reference beads has the potential to help mitigate those variations, thereby increasing the accuracy of simultaneous assays.

Most broadly, the present study demonstrates the viability of multiplexed assays based on holographic characterization of multiple classes of test beads codispersed in the same test kit. The present implementation dilutes the analyte from physiologically relevant concentrations down to levels suitable for bead-based binding assays. Other combinations of analyte volume and initial bead concentration can be used to provide greater sensitivity at the expense of a more limited range of accessible concentrations. Demonstrations of such concentration-optimized multitarget multiplexed assays will be reported separately.
